# Supplementary material for: Continuous population-level monitoring of SARS-CoV-2 seroprevalence in a large European metropolitan region
Source: iScience. 2023 Jan 4;26(2):105928. doi: 10.1016/j.isci.2023.105928 (PMC9811913; doi:10.1016/j.isci.2023.105928)
Supplement: Document S1. Figures S1–S9 and Tables S1–S5 [file mmc1.pdf]

iScience, Volume *26*

## Supplemental information

# Continuous population-level monitoring of SARS-CoV-2 seroprevalence in a large European metropolitan region

**Marc Emmenegger, Elena De Cecco, David Lamparter, Raphaël P.B. Jacquat, Julien Riou, Dominik Menges, Tala Ballouz, Daniel Ebner, Matthias M. Schneider, Itzel Condado Morales, Berre Doğançay, Jingjing Guo, Anne Wiedmer, Julie Domange, Marigona Imeri, Rita Moos, Chryssa Zografou, Leyla Batkitar, Lidia Madrigal, Dezirae Schneider, Chiara Trevisan, Andres Gonzalez-Guerra, Alessandra Carrella, Irina L. Dubach, Catherine K. Xu, Georg Meisl, Vasilis Kosmoliaptsis, Tomas Malinauskas, Nicola Burgess-Brown, Ray Owens, Stephanie Hatch, Juthathip Mongkolsapaya, Gavin R. Screaton, Katharina Schubert, John D. Huck, Feimei Liu, Florence Pojer, Kelvin Lau, David Hacker, Elsbeth Probst-Müller, Carlo Cervia, Jakob Nilsson, Onur Boyman, Lanja Saleh, Katharina Spanaus, Arnold von Eckardstein, Dominik J. Schaer, Nenad Ban, Ching-Ju Tsai, Jacopo Marino, Gebhard F.X. Schertler, Nadine Ebert, Volker Thiel, Jochen Gottschalk, Beat M. Frey, Regina R. Reimann, Simone Hornemann, Aaron M. Ring, Tuomas P.J. Knowles, Milo A. Puhan, Christian L. Althaus, Ioannis Xenarios, David I. Stuart, and Adriano Aguzzi**

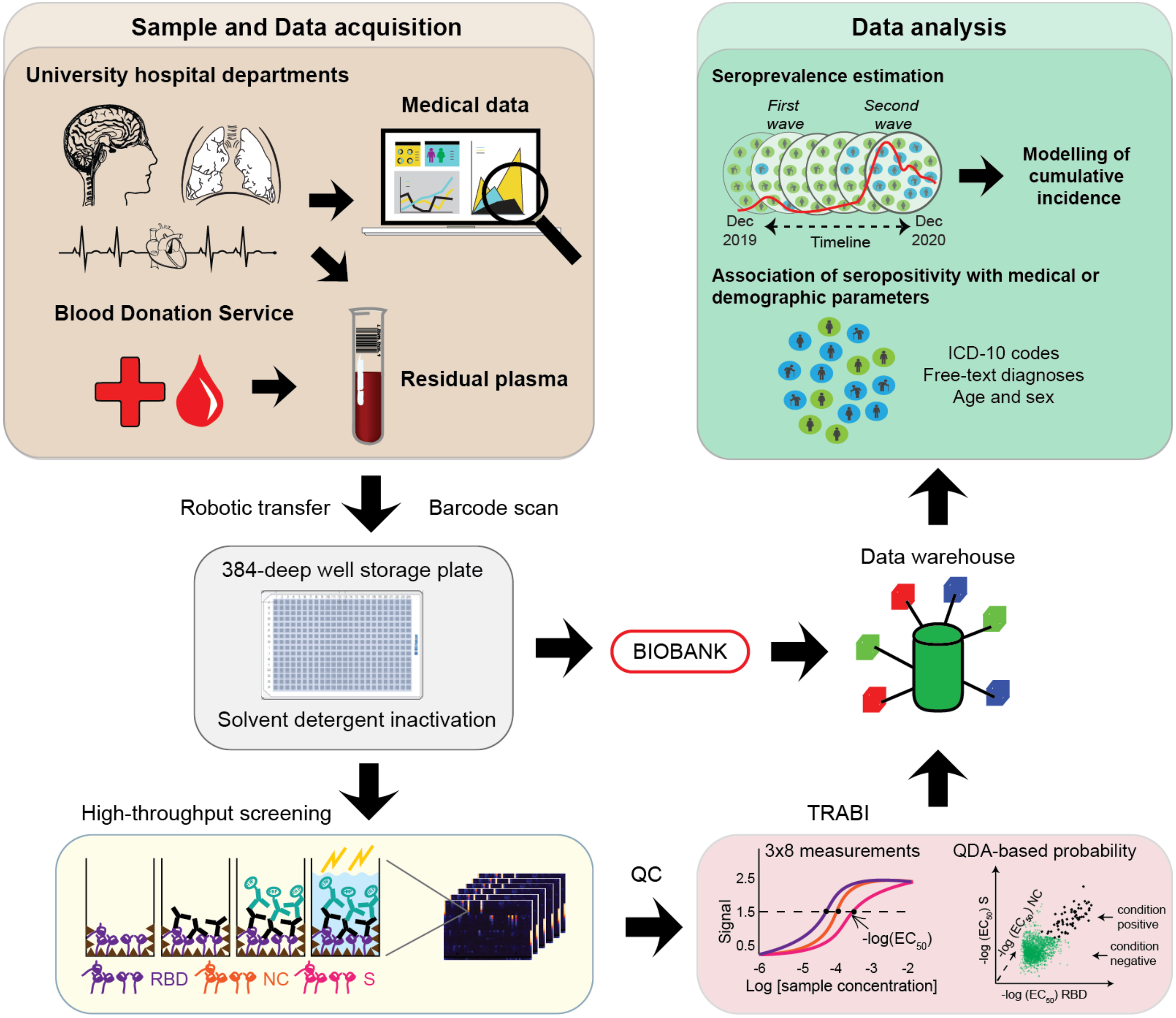


**Fig. S1**. **Detailed assay procedure, related to Fig. 1A**. Barcoded residual plasma samples from patients admitted to almost all clinical departments of the University Hospital Zurich (USZ) or from healthy donors donating blood to the Blood Donation Services (BDS) Canton Zurich were robotically transferred into 384-deep well plates and subjected to viral inactivation using a solvent detergent procedure. The plates were subsequently stored in a liquid high-throughput biobank, while associated medical data were stored in a custom-designed data warehouse. The samples were used to conduct high-throughput serological assays to interrogate for the presence of antibodies against multiple CoV2 proteins, with an indirect ELISA. To this end, plates were coated with S, RBD, or NC. Following the mapping of ELISA results, antibody titers (i.e. -log($EC_{50}$)) against the three antigens were determined from eight sample dilutions via logistic regression. The -log($EC_{50}$) profiles from both condition negatives (prepandemic samples) and condition positives (convalescent CoV2-PCR-confirmed individuals for BDS and COVID-19 patients for USZ) were used to infer the QDA-based posterior probability of each sample to be seropositive and data was stored in afore mentioned data warehouse. The data was then used for seroprevalence estimation and the modelling of the cumulative incidence. Medical and demographic data was used to interrogate the presence of potential associations between disease and CoV2 positivity.

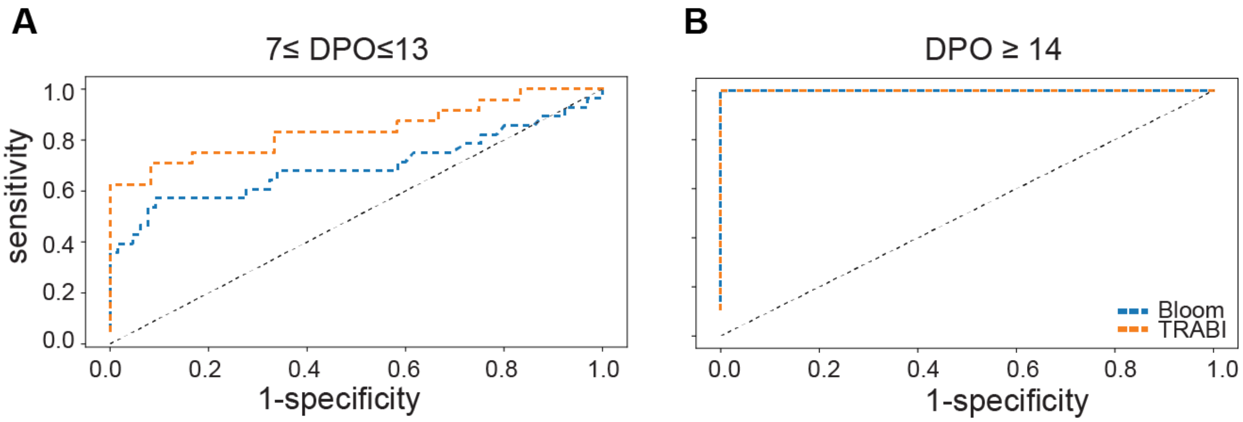


**Fig. S2**. **Comparison of TRABI to a lateral-flow assay developed by Bloom diagnostics, related to Fig. 1C**. **A**. Comparison between the bloom test and TRABI of samples taken 7 ≤ DPO ≤ 13. **B**. Comparison between the bloom test and TRABI of samples taken DPO ≥ 14.

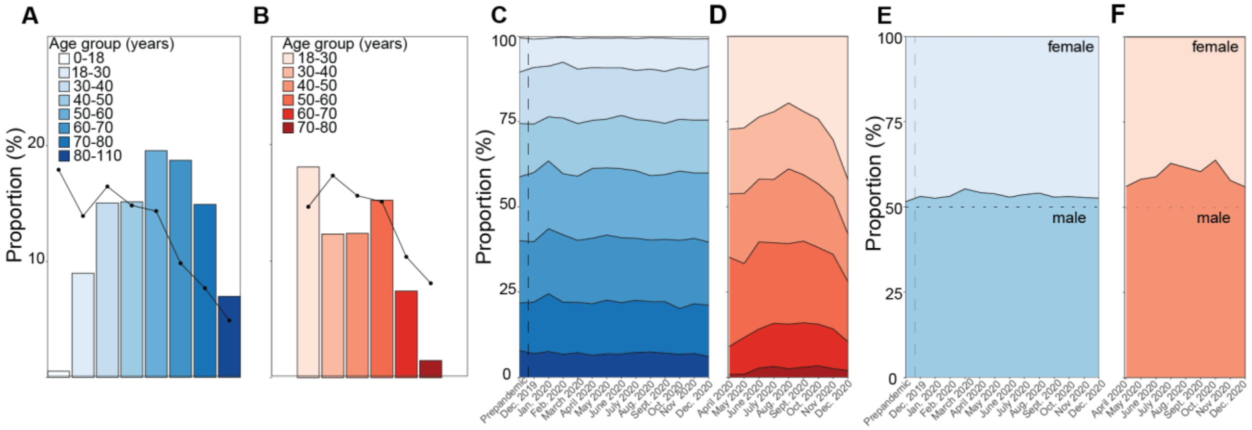


**Fig. S3**. **Age and sex characteristics of the two cohorts, related to Figs. 2 and 3**. **A** and **B**. Sample distribution according to age groups for USZ (A) and for BDS (B). The age distribution in the USZ sample is quite different from the general population of canton Zürich (black dots). **C** and **D**. Proportion of age groups over time for USZ (C) and for BDS (D). **E** and **F**. Sex distribution over time for USZ (E) and for BDS (F). If there are multiple samples per patient (but never within the same month, as outlined), multiple samples pertaining to the same patient have been included in the analysis.

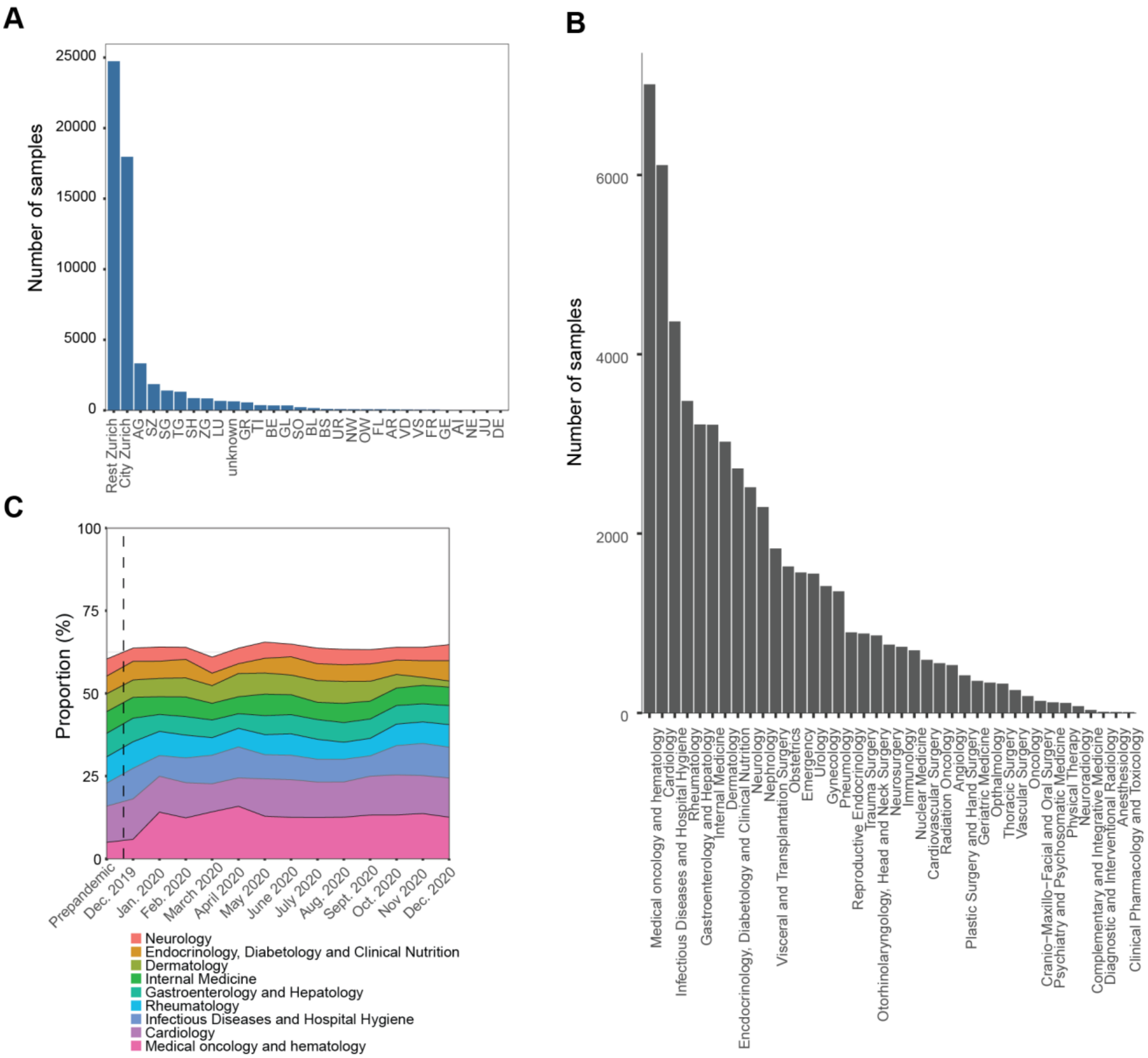


**Fig. S4**. **Sample provenance and clinical wards of the USZ patients, related to Figs. 2 and 3**. **A**. Sample origin for USZ patients. The two letter codes are official abbreviations of the cantons in Switzerland. Most USZ patients are from the city or from the surroundings of Zurich, followed by the neighbouring cantons. **B**. Clinical departments at which patients were treated. **C**. Distribution of clinical departments over time of study. Except for one department, Medical oncology and hematology, which shows an increase from prepandemic times to January 2020, likely owing to re-structuring of the department, the provenance was stable over time.

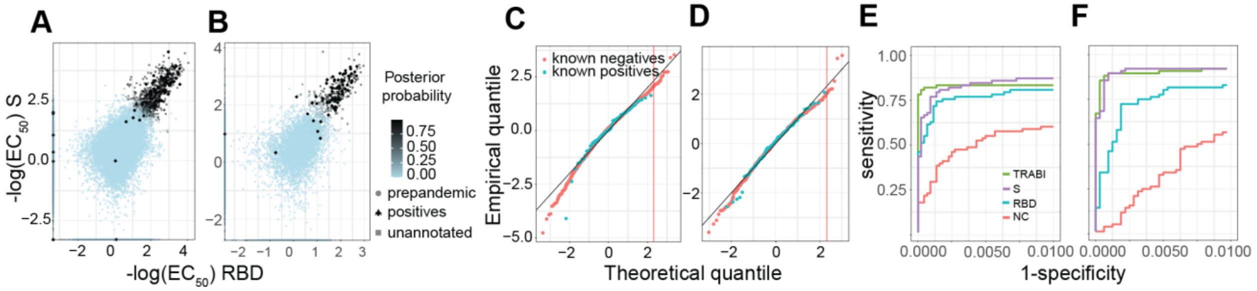


**Fig. S5**. **Prevalence estimation in two large cohorts, related to Fig. 2**. **A.** and **B**. Depicted are all the –log($EC_{50}$) values calculated for S and the RBD for the USZ (A) and the BDS (B) cohort. Posterior probabilities were calculated using LDA. **C**. and. **D**. Q-Q plots to check the Gaussian distributional assumption of the LDA model. For USZ (C) and the BDS (D) cohort respectively, we collapsed the 3 measures per sample according to the linear discriminant classifier. We then scaled known positives and negatives to mean zero and unit variance and compared their distributions to the univariate Gaussian distribution via Q-Q plot. We saw that for the relevant upper tail, the distribution of known negatives followed the normal distribution with only a mild deviation, such that, for instance, the upper one percent quantile is reached at 2.07 (C) and 1.99 (D) rather than at their theoretical value of 2.33. **E**. and. **F**. ROC curves for the USZ (E) and BDS (F) cohorts using the prepandemic samples (including condition negatives from December 2019 and January 2020 for BDS) as condition negatives and selected condition positives from both cohorts.

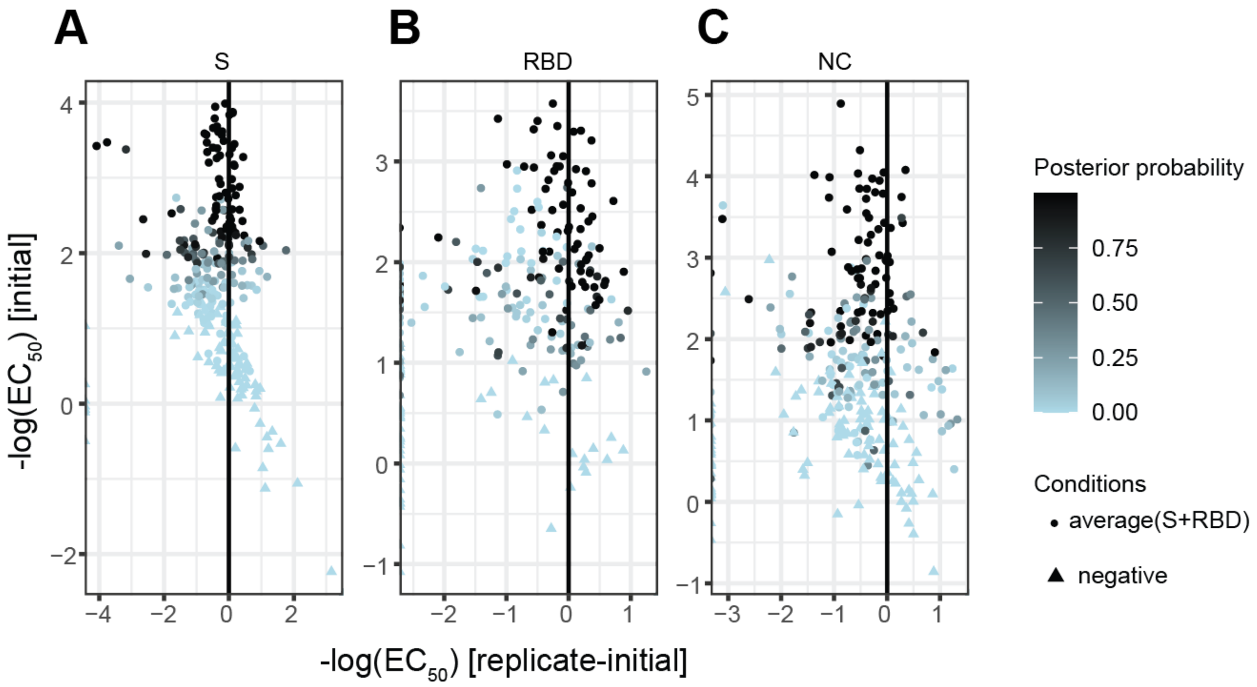


**Fig. S6. Assay reproducibility using 210 high scoring samples and 122 random samples (based on results from the high-throughput screen) for binding against S, the RBD, and the NC, related to Figs. 2 and 3**. **A.** S binding shows that reproducibility increases at higher values, consistent with increased posterior probabilities. **B.** Same observation as (A) for the RBD. **C**. Same observation as (A) for the NC.

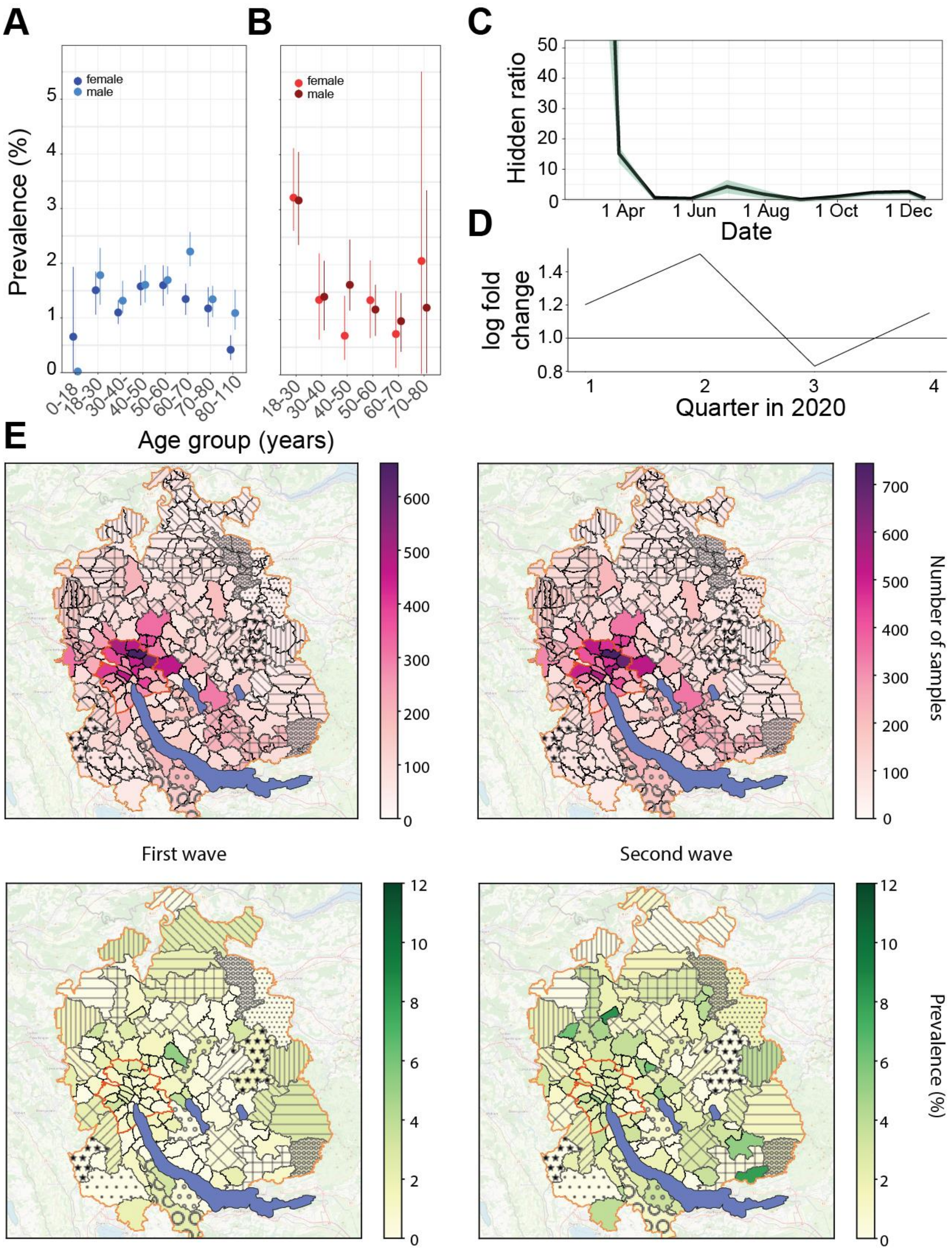


**Fig. S7. Epidemiological modelling, related to Figs. 3 and 4**. **A**. Seroprevalence according to age and sex for the hospital patients. **B**. Seroprevalence according to age and sex for the blood donors. **C**. Hidden epidemic ratio. **D**. Log fold change of seropositivity of hospital patients from the city versus non-city in four quarters, covering the first and the second wave. **E**. We display the same maps as shown in **Fig. 4** after forming arbitrary groups of zip codes to increase the sample size per region. Hence, we circumvent the need to exclude areas where the total counts were less than 50 patients. The maps display a similar picture as shown in **Fig. 4**, indicating that limiting the analysis to areas with at least 50 patients in total is not posing a representation bias.

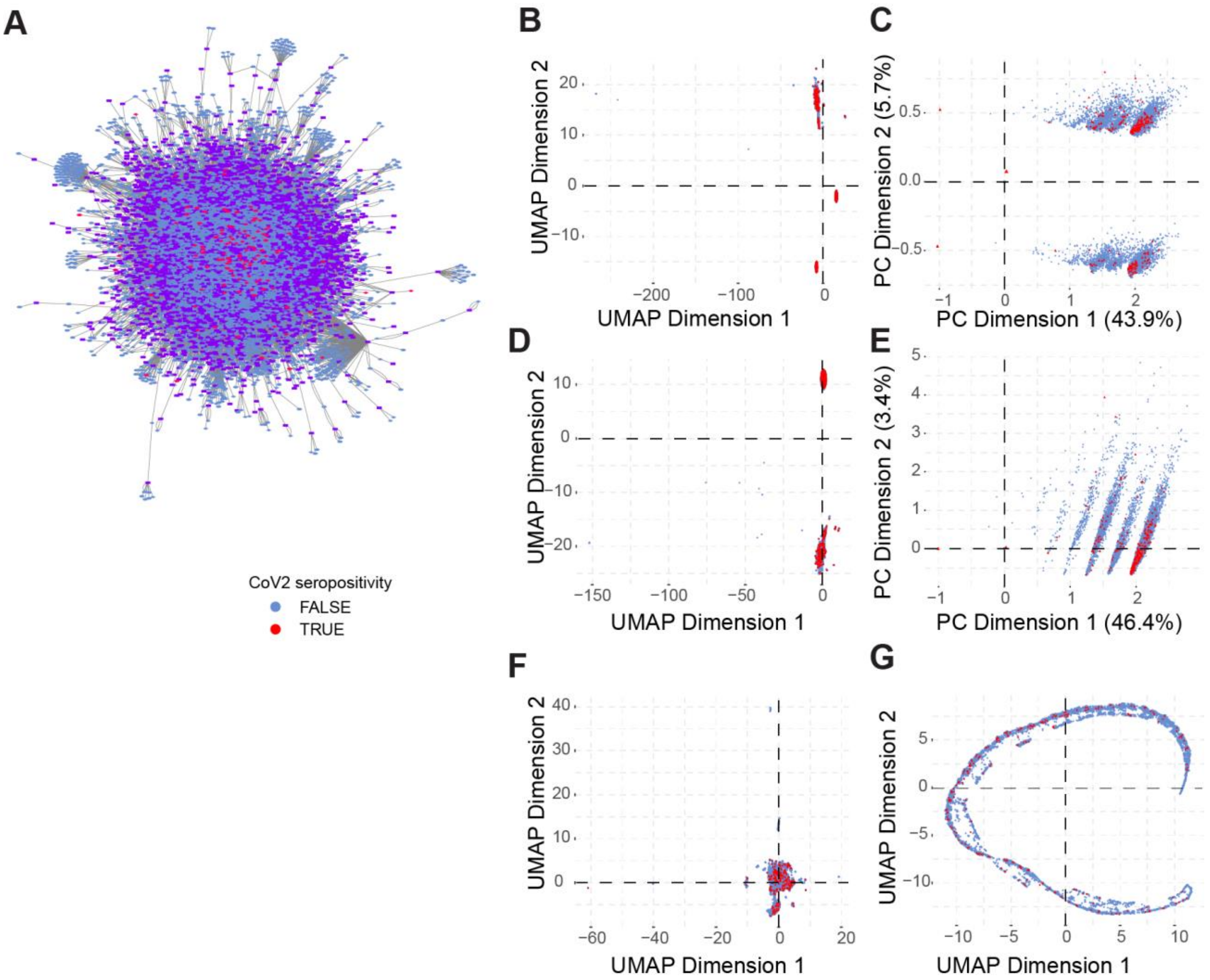


**Fig. S8. Exploratory analysis of CoV2 seropositivity with ICD-10 codes and free-text medical reports, related to Fig. 5**. **A**. Force directed layout of topological network. ICD-10 codes: purple rectangles. Male patients: diamonds, female patients: circles. Seropositive: red. Seronegative: blue. **B**. Two-dimensional projections of ICD-10 codes as well as female and male sex following UMAP tuned for binary data input using cosine metric. **C**. Principal component analysis of ICD-10 codes as well as female and male sex. **D**. and **E**. Same as in (B) and (C) with exclusion of male/female sex as feature. **F**. and **G**. Representation of dataset with exclusion of patients for which no ICD-10 data exists, using cosine (F) or Euclidean (G) distances. For A-G, seropositivity: red. Seronegativity: blue. No distributional differences between seropositive and seronegative hospital patients were visible.

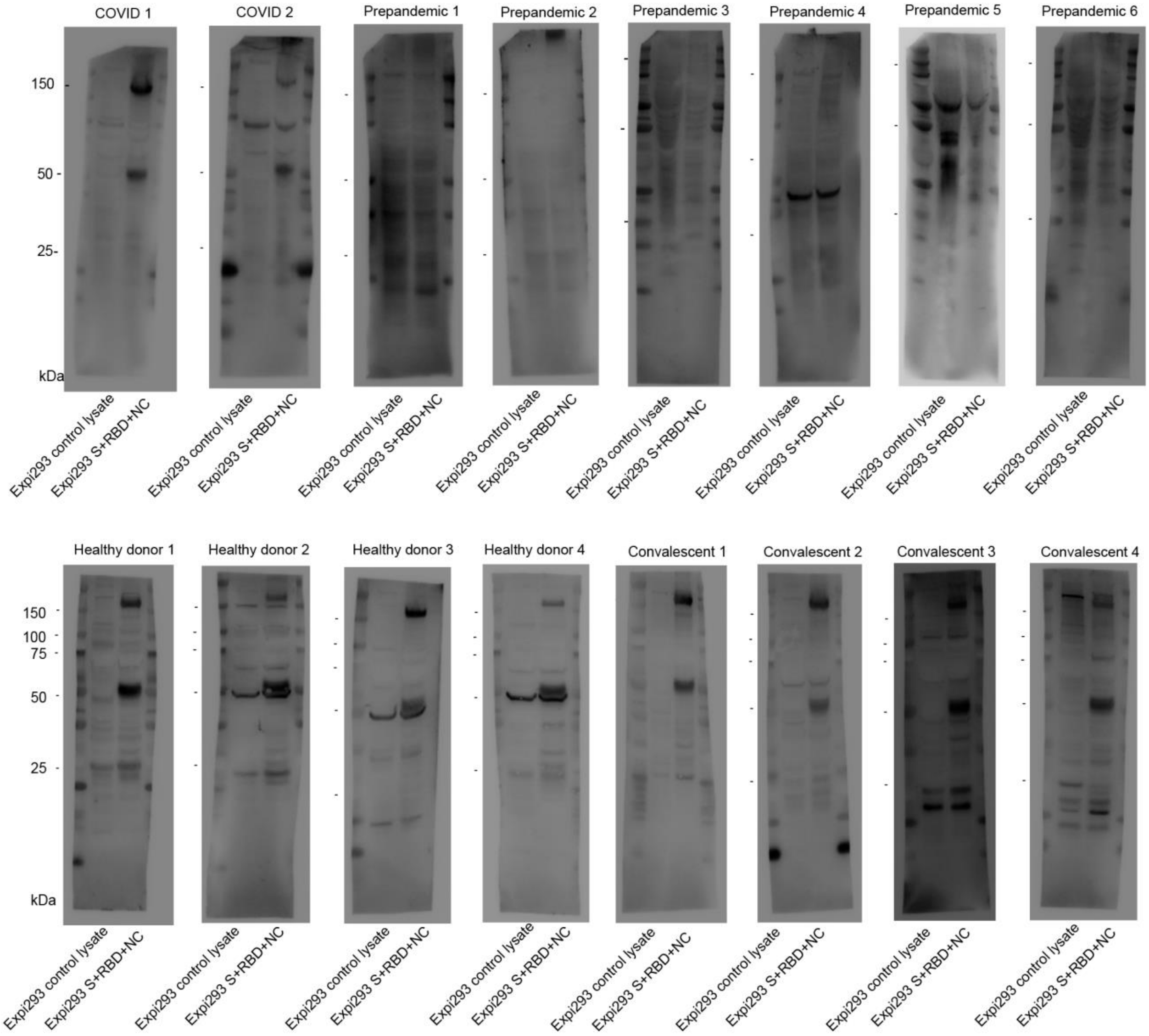


**Fig. S9. Uncropped and unmodified camera-acquired images of the Western Blots, related to Figs. 6 and 7.**

| | Total | No oxygen supply | Oxygen supply | Intubation |
|---|---|---|---|---|
| Individuals, number | 27 | 8 | 12 | 7 |

**Table S1, related to Fig. 1 and Table 1. Characterization of cohort used for assay establishment in terms of disease severity.**

| ICD-10 | ICD-10 Text | Nr. of patients with code |
|---|---|---|
| I10.00 | Essentielle (primäre) Hypertonie | 3251 |
| U99.0 | Spezielle Verfahren zur Untersuchung auf SARS-CoV-2 | 2790 |
| Z11 | Spezielle Verfahren zur Untersuchung auf infektiöse und parasitäre Krankheiten | 2773 |
| Y84.9 | Zwischenfälle durch medizinische Maßnahmen, nicht näher bezeichnet | 1756 |
| Z92.1 | Dauertherapie (gegenwärtig) mit Antikoagulanzien in der Eigenanamnese | 1615 |
| N18.3 | Chronische Nierenkrankheit, Stadium 3 | 1259 |
| I48.0 | Vorhofflimmern, paroxysmal | 1218 |
| D90 | Immunkompromittierung nach Bestrahlung, Chemotherapie und sonstigen immunsuppressiven Maßnahmen | 1184 |
| U69.12 | Temporäre Blutgerinnungsstörung | 1096 |
| B96.2 | Escherichia coli [E. coli] und andere Enterobacterales als Ursache von Krankheiten, die in anderen Kapiteln klassifiziert sind | 1072 |
| Z86.7 | Krankheiten des Kreislaufsystems in der Eigenanamnese | 1001 |
| Z95.5 | Vorhandensein eines Implantates oder Transplantates nach koronarer Gefäßplastik | 992 |
| E11.90 | Diabetes mellitus, Typ 2, mit Koma | 904 |
| I25.13 | Drei-Gefäß-Erkrankung | 828 |
| D69.58 | Sonstige sekundäre Thrombozytopenien, nicht als transfusionsrefraktär bezeichnet | 820 |
| E55.9 | Vitamin-D-Mangel, nicht näher bezeichnet | 816 |
| E78.4 | Sonstige Hyperlipidämien | 795 |
| D62 | Akute Blutungsanämie | 793 |
| Y82.8 | Zwischenfälle durch medizintechnische Geräte und Produkte | 776 |
| E87.6 | Hypokaliämie | 734 |
| F05.8 | Sonstige Formen des Delirs | 722 |
| Y57.9 | Komplikationen durch Arzneimittel oder Drogen | 711 |
| E03.8 | Sonstige näher bezeichnete Hypothyreose | 673 |
| I50.01 | Rechtsherzinsuffizienz, Sekundäre Rechtsherzinsuffizienz | 656 |
| Z92.2 | Dauertherapie (gegenwärtig) mit anderen Arzneimitteln in der Eigenanamnese | 653 |
| J96.00 | Akute respiratorische Insuffizienz, anderenorts nicht klassifiziert | 638 |
| I50.12 | Linksherzinsuffizienz, Mit Beschwerden bei stärkerer Belastung | 629 |
| I25.11 | Ein-Gefäß-Erkrankung | 619 |
| N39.0 | Harnwegsinfektion, Lokalisation nicht näher bezeichnet | 614 |
| I25.12 | Zwei-Gefäß-Erkrankung | 606 |
| X59.9 | Sonstiger und nicht näher bezeichneter Unfall | 597 |
| D68.4 | Erworbener Mangel an Gerinnungsfaktoren | 589 |
| N40 | Prostatahyperplasie | 570 |
| I10.90 | Essentielle Hypertonie, nicht näher bezeichnet | 566 |
| C43.9 | Bösartiges Melanom der Haut, nicht näher bezeichnet | 551 |
| E78.8 | Sonstige Störungen des Lipoproteinstoffwechsels | 547 |
| E87.1 | Hypoosmolalität und Hyponatriämie | 531 |
| I34.0 | Mitralklappeninsuffizienz | 526 |
| I11.90 | Hypertensive Herzkrankheit ohne (kongestive) Herzinsuffizienz | 507 |
| Z95.88 | Vorhandensein von sonstigen kardialen oder vaskulären Implantaten oder Transplantaten | 504 |
| E11.91 | Diabetes mellitus, Typ 2, mit Ketoazidose | 493 |
| E83.38 | Sonstige Störungen des Phosphorstoffwechsels und der Phosphatase | 493 |
| U69.00 | Anderenorts klassifizierte, im Krankenhaus erworbene Pneumonie | 491 |
| I50.14 | Linksherzinsuffizienz, mit Beschwerden in Ruhe | 476 |
| I50.13 | Linksherzinsuffizienz, mit Beschwerden bei leichterer Belastung | 472 |
| D50.8 | Sonstige Eisenmangelanämien | 467 |
| E78.0 | Reine Hypercholesterinämie | 455 |
| D64.8 | Sonstige näher bezeichnete Anämien | 445 |
| J91 | Pleuraerguss bei anderenorts klassifizierten Krankheiten | 443 |
| A49.8 | Sonstige bakterielle Infektionen nicht näher bezeichneter Lokalisation | 438 |

**Table S2, related to Table 1 and Fig. 5. 50 most common ICD-10 codes for hospital patients registered in hospital-wide database.**

| | Seronegative | Seropositive | Overall |
|---|---|---|---|
| Individuals, number | 65 | 71 | 136 |
| ***Infected prior to sampling*** | | | |
| Yes | 1 (1.6%) | 38 (53.5%) | 39 (28.9%) |
| No | 63 (98.4%) | 33 (46.5%) | 96 (71.1%) |
| Missing | 1 (1.5%) | 0 (0%) | 1 (0.7%) |
| | | | |
| ***Infected in 2020*** | | | |
| Yes | 3 (10.7%) | 42 (80.8%) | 45 (56.2%) |
| No | 25 (89.3%) | 10 (19.2%) | 35 (43.8%) |
| Missing | 37 (56.9%) | 19 (26.8%) | 56 (41.2%) |
| | | | |
| ***Ever infected*** | | | |
| Yes | 28 (44.4%) | 52 (77.6%) | 80 (61.5%) |
| No | 34 (54.0%) | 14 (20.9%) | 48 (36.9%) |
| Don't know | 1 (1.6%) | 1 (1.5%) | 2 (1.5%) |
| Missing | 2 (3.1%) | 4 (5.6%) | 6 (4.4%) |
| | | | |
| ***Ever reinfected*** | | | |
| Yes | 0 (0.0%) | 11 (21.2%) | 11 (13.8%) |
| No | 27 (96.4%) | 40 (76.9%) | 67 (83.8%) |
| Don't know | 1 (3.6%) | 1 (1.9%) | 2 (2.5%) |
| Missing | 37 (56.9%) | 19 (26.8%) | 56 (41.2%) |

**Table S3, related to Fig. 5. Knowledge of infection prior to blood sampling among online health survey participants tested seropositive or seronegative.**

| | Spring/Summer 2020 | Fall/Winter 2020/2021 | Winter/Spring 2021/2022 | Overall |
|---|---|---|---|---|
| Individuals, number | 12 | 34 | 34 | 80 |
| ***Symptoms at infection*** | | | | |
| Yes | 10 (90.9%) | 25 (73.5%) | 29 (85.3%) | 64 (81.0%) |
| No | 1 (9.1%) | 9 (26.5%) | 5 (14.7%) | 15 (19.0%) |
| Missing | 1 (8.3%) | 0 (0%) | 0 (0%) | 1 (1.3%) |
| | | | | |
| ***Hospitalisation due to COVID-19*** | | | | |
| Yes | 5 (41.7%) | 8 (23.5%) | 1 (2.9%) | 14 (17.5%) |
| No | 7 (58.3%) | 26 (76.5%) | 33 (97.1%) | 66 (82.5%) |
| | | | | |
| ***Oxygen supplementation*** | | | | |
| Yes | 4 (80.0%) | 6 (75.0%) | 1 (100.0%) | 11 (78.6%) |
| No | 1 (20.0%) | 2 (25.0%) | 0 (0.0%) | 3 (21.4%) |
| Missing | 7 (58.3%) | 26 (76.5%) | 33 (97.1%) | 66 (82.5%) |
| | | | | |
| ***Intensive care treatment*** | | | | |
| Yes | 4 (80.0%) | 3 (37.5%) | 1 (100.0%) | 8 (57.1%) |
| No | 1 (20.0%) | 5 (62.5%) | 0 (0.0%) | 6 (42.9%) |
| Missing | 7 (58.3%) | 26 (76.5%) | 33 (97.1%) | 66 (82.5%) |
| | | | | |
| ***Mechanical ventilation*** | | | | |
| Yes | 2 (50.0%) | 3 (100.0%) | 1 (100.0%) | 6 (75.0%) |
| No | 2 (50.0%) | 0 (0.0%) | 0 (0.0%) | 2 (25.0%) |
| Missing | 8 (66.7%) | 31 (91.2%) | 33 (97.1%) | 72 (90.0%) |
| | | | | |
| ***Diagnosed pneumonia*** | | | | |
| Yes | 3 (25.0%) | 5 (15.2%) | 1 (3.0%) | 9 (11.5%) |
| No | 8 (66.7%) | 28 (84.8%) | 29 (87.9%) | 65 (83.3%) |
| Don't know | 1 (8.3%) | 0 (0.0%) | 3 (9.1%) | 4 (5.1%) |
| Missing | 0 (0%) | 1 (2.9%) | 1 (2.9%) | 2 (2.5%) |

**Table S4, related to Fig. 5. Severity of infection among online health survey participants reporting a known CoV2 infection up to April/May 2022, stratified by infection wave.**

| | Never infected | Infected | Overall |
|---|---|---|---|
| Individuals, number | 50 | 80 | 136 |
| ***EQ-5D-5L score*** | | | |
| Mean (SD) | 0.81 (0.17) | 0.87 (0.19) | 0.85 (0.18) |
| Median (IQR) | 0.85 (0.74 to 1.00) | 0.91 (0.83 to 1.00) | 0.89 (0.79 to 1.00) |
| Range | 0.42 to 1.00 | -0.02 to 1.00 | -0.02 to 1.00 |
| Missing | 5 (10.0%) | 11 (13.8%) | 20 (14.7%) |
| | | | |
| ***EQ VAS score*** | | | |
| Mean (SD) | 70.30 (20.88) | 75.00 (15.83) | 72.92 (18.18) |
| Median (IQR) | 74.50 (60.75 to 89.50) | 78.50 (70.25 to 84.75) | 76.00 (70.00 to 85.00) |
| Range | 18.00 to 97.00 | 10.00 to 100.00 | 10.00 to 100.00 |
| Missing | 4 (8.0%) | 10 (12.5%) | 18 (13.2%) |

**Table S5, related to Fig. 5. Health status measured by EQ-5D-5L and EQ VAS among online health survey participants reporting a known CoV2 infection up to April/May 2022.**
